# Disseminated *Mycobacterium kansasii* infection with osseous involvement in anti-interferon-γ autoantibody-associated adult-onset immunodeficiency: a case report and literature review

**DOI:** 10.3389/fimmu.2026.1841472

**Published:** 2026-06-23

**Authors:** Jian Wang, Keying Lin, Yidan Zhong, Zhiyu Wu, Tao Lu, WeiLi Lu, Weiguo Wang, Cuifang Ma

**Affiliations:** 1 Department of Gastroenterology, Jiaxing Second Hospital, Jiaxing, Zhejiang, China; 2 Zhejiang Chinese Medical University, Hangzhou, Zhejiang, China; 3 Department of Infectious Diseases, The Quzhou Affiliated Hospital of Wenzhou Medical University, Quzhou People’s Hospital, Quzhou, Zhejiang, China

**Keywords:** anti-interferon-γ autoantibody, bone involvement, case report, disseminated infection, *Mycobacterium kansasii*

## Abstract

**Background:**

Anti–interferon-γ autoantibody-associated adult-onset immunodeficiency is a rare acquired immunodeficiency that predisposes patients to recurrent or disseminated opportunistic infections, particularly nontuberculous mycobacterial (NTM) infections. Disseminated *Mycobacterium kansasii* infection in this setting is uncommon and may radiologically mimic malignancy, leading to diagnostic delay.

**Case presentation:**

A 53-year-old Chinese man with untreated chronic hepatitis B virus (HBV) infection presented with cough, chest pain, and back pain. Chest computed tomography and ^18F-FDG PET/CT revealed a left hilar mass, mediastinal and hilar lymphadenopathy, and extensive FDG-avid skeletal lesions, initially suggesting lung cancer with bone metastases. However, repeated pathological examinations, including bronchoscopic brushing, endobronchial ultrasound-guided transbronchial needle aspiration, and cervical lymph node aspiration, failed to confirm malignancy. Targeted next-generation sequencing of bronchoalveolar lavage fluid and metagenomic next-generation sequencing of vertebral tissue both identified *Mycobacterium kansasii*, supporting disseminated infection with pulmonary and skeletal involvement. Subsequent immunologic testing demonstrated elevated anti–IFN-γ autoantibodies, supporting a clinical diagnosis of AIGA-associated disseminated *M. kansasii* infection. Antimycobacterial therapy was initiated, but further treatment was complicated by postoperative cholestatic jaundice and high-level HBV viremia, which precluded immediate escalation to immune-directed therapy.

**Conclusions:**

AIGA-associated disseminated *Mycobacterium kansasii* infection can closely mimic lung cancer with bone metastases. In patients with tumor-like pulmonary and skeletal lesions but repeatedly nondiagnostic pathology, early integration of pathogen detection and anti–IFN-γ autoantibody testing may help shorten diagnostic delay.

## Introduction

Anti–interferon-γ autoantibody-associated adult-onset immunodeficiency is a rare acquired immune disorder characterized by anti–IFN-γ autoantibodies (AIGA), which impair IFN-γ–mediated cellular immunity (1, 2). Clinically, it presents with recurrent or disseminated opportunistic infections involving multiple organs, often following a prolonged and relapsing course. Reported pathogens include nontuberculous mycobacteria (NTM), *Mycobacterium tuberculosis*, *Salmonella* species, and varicella–zoster virus (VZV), among which NTM infections predominate (3).

In the spectrum of AIGA-associated NTM infections, Mycobacterium abscessus and the Mycobacterium avium complex (MAC) are more frequently identified, whereas *Mycobacterium kansasii (M. kansasii)* accounts for a smaller proportion (4, 5). Recent bibliometric analyses indicate that Mycobacterium kansasii comprises approximately 10% of NTM infections in AIGA patients (5). When disseminated, AIGA-associated NTM infection may involve the lymph nodes, skin, musculoskeletal system, and genitourinary system. The imaging features of AIGA-associated NTM infection are often nonspecific, and in some cases may mimic malignant diseases such as lung cancer, thereby contributing to misdiagnosis or diagnostic delay. Wu et al. reported a misdiagnosis rate of approximately 33%, with cases frequently mistaken for tuberculosis, metastatic malignancy, connective tissue disorders, or lymphoma (6). Here, we report a case of AIGA-associated disseminated *M. kansasii* infection involving the lung and skeleton, in which the imaging findings closely resembled primary lung cancer with multiple bone metastases. Repeated pathological examinations failed to demonstrate malignancy, and the final diagnosis was established only after next-generation sequencing and immunologic testing. This case highlights the diagnostic challenge posed by disseminated NTM infection in adults without previously recognized immunodeficiency and underscores the importance of considering AIGA when invasive pathology remains inconclusive despite strong radiologic suspicion of malignancy.

## Case presentation

**Figure 1:**
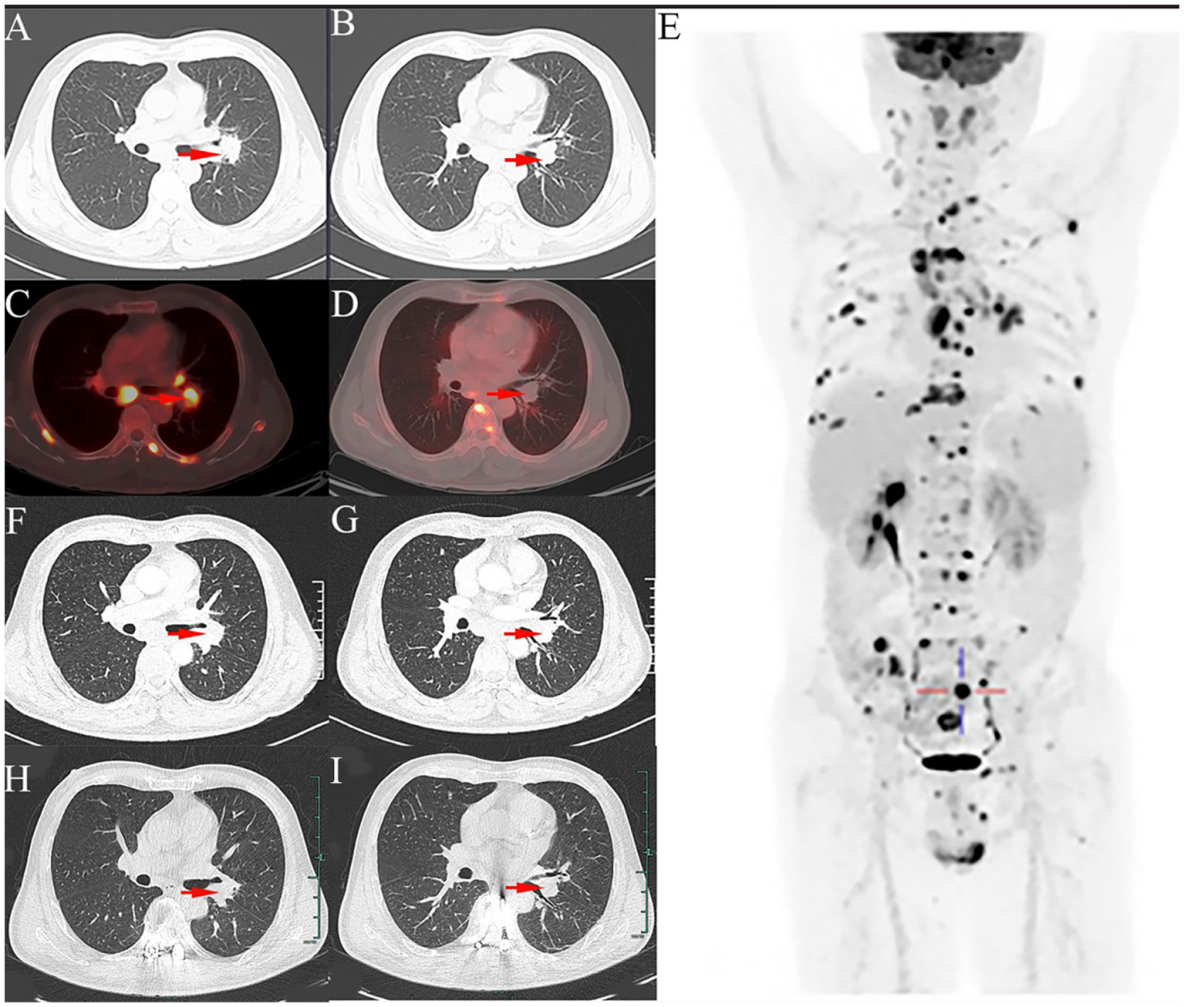
Dynamic radiologic changes of the left hilar/left upper-lobe mass and systemic FDG uptake during the clinical course (red arrows). **(A, B)**, April 2025 (contrast-enhanced chest CT). **(C, D)**, May 15, 2025 (^18F-FDG PET). **(E)**, May 15, 2025 (^18F-FDG PET maximum intensity projection showing disseminated FDG-avid lesions). **(F, G)**, July 2025. **(H, I)**, January 2026.

**Figure 2:**
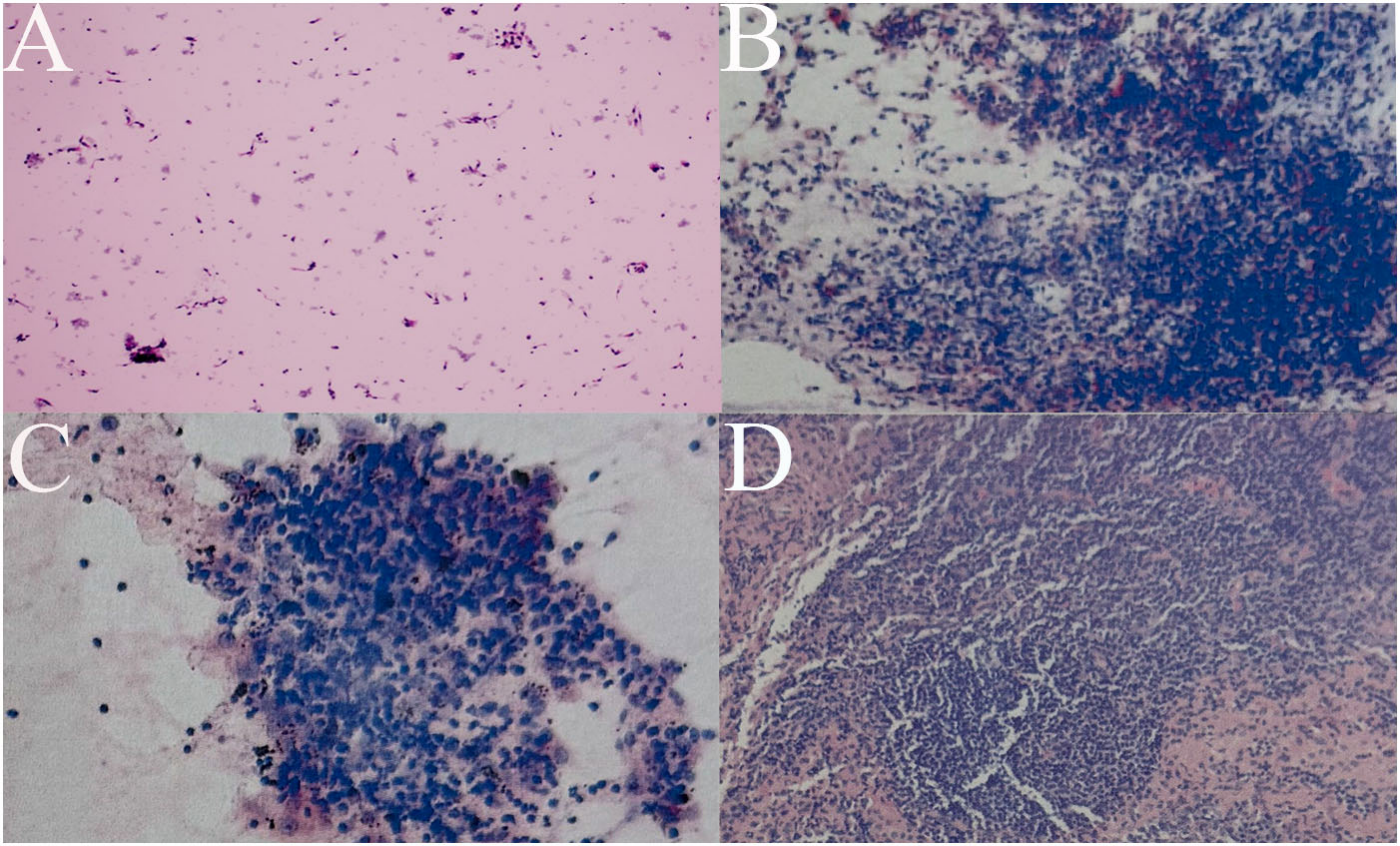
Cytologic and pathologic findings from repeated malignancy-directed sampling. **(A)**, Bronchial brushing cytology in April 2025 showing ciliated columnar epithelial cells and scattered inflammatory cells, without malignant cells (Papanicolaou stain; original magnification ×400). **(B)**, Repeat bronchial brushing cytology on May 23, 2025 showing no malignant cells (Papanicolaou stain; original magnification ×400). **(C)**, EBUS-TBNA specimen from mediastinal lymph node station 7 showing lymphoid cellular material without malignant epithelial cells or atypical lymphoid proliferation (liquid-based cytology, Papanicolaou stain; original magnification ×400). **(D)**, Right cervical lymph node specimen showing chronic lymphadenitis with fibrosis, without granulomatous inflammation, necrosis, or atypical lymphoid proliferation (H&E stain; original magnification ×100).

A 53-year-old Chinese man presented to an outside hospital in April 2025 with a 1-month history of chest and back pain accompanied by cough. He denied smoking, alcohol use, and a relevant family history. His medical history was notable for chronic hepatitis B virus infection for more than 20 years, without prior antiviral therapy. Screening for common immunocompromising conditions, including HIV infection, diabetes mellitus, liver cirrhosis, and prior immunosuppressive therapy, was unremarkable. Contrast-enhanced chest computed tomography (CT) revealed a left hilar mass suspicious for primary lung malignancy with possible mediastinal lymph node involvement (**Figures 1A, B**). Bronchial brushing cytology was negative for malignancy and showed only ciliated columnar epithelial cells with scant inflammatory cells (**Figure 2A**). He was subsequently referred to our hospital for further evaluation. On May 15, 2025, ^18F-FDG PET/CT demonstrated a hypermetabolic lesion in the left upper lobe with segmental bronchial narrowing and partial obstruction, strongly suggesting lung cancer (**Figures 1C, D**). Multiple FDG-avid mediastinal and hilar lymph nodes were also identified, raising concern for nodal metastases. In addition, numerous FDG-avid osseous lesions involving the skull, spine, ribs, sternum, scapulae, pelvis, and left femur were observed, consistent with extensive skeletal metastatic disease, and a pathologic fracture of the T3 vertebral body was noted (**Figure 1E**). A focal mildly FDG-avid splenic lesion was also detected and considered suspicious for metastasis. Based on these findings, the initial working diagnosis was primary lung malignancy with multiple metastases.

To further clarify the diagnosis, repeat bronchoscopic brushing was performed on May 23, 2025, from the apicoposterior segment of the left upper lobe, but again showed no malignant cells (**Figure 2B**). Endobronchial ultrasound (EBUS) revealed a hypoechoic lesion at mediastinal lymph node station 7, and liquid-based cytology from EBUS-guided transbronchial needle aspiration (EBUS-TBNA) likewise showed no evidence of malignancy (**Figure 2C**). Fine-needle aspiration of a right cervical lymph node demonstrated chronic lymphadenitis with fibrosis (**Figure 2D**). Further evaluation was temporarily interrupted because the patient declined continued hospitalization for financial reasons.

In July 2025, he was readmitted because of recurrent cough. Repeat bronchial brushing and cytologic examination of an aspirate from mediastinal lymph node station 7 again failed to identify definite malignant cells. A repeat contrast-enhanced chest CT showed persistent left hilar soft-tissue mass-like shadowing with mediastinal and hilar lymphadenopathy, without substantial change from prior imaging (**Figures 1F, G**). Given the repeated negative pathological results despite radiologic suspicion of malignancy, bronchoalveolar lavage fluid was submitted for targeted next-generation sequencing (tNGS) on July 28, 2025. The assay was performed by WillingMed (Beijing, China), using the PathoXtract WYXM03010S mcfDNA enrichment extraction kit and the MGISEQ-200 platform. The assay detected Mycobacterium kansasii (8,057 reads), along with Pneumocystis jirovecii (335 reads), Streptococcus pneumoniae (3,211 reads), Burkholderia neonosa (186 reads), and Epstein-Barr virus (11,021 reads). In the context of the patient’s clinical presentation and imaging findings, *M. kansasii* was considered the most plausible pathogen. Because no culture isolate was available for drug susceptibility testing, antimycobacterial therapy was empirically initiated according to guideline-based treatment principles, with isoniazid 300 mg once daily, rifampin 0.6 g once daily, and ethambutol 0.5 g once daily. During treatment, his cough partially improved, but intermittent chest discomfort persisted. The white blood cell count and C-reactive protein level remained within the normal range both at treatment initiation and when therapy was later withheld.

In December 2025, the patient was readmitted because of worsening low back pain. Metagenomic next-generation sequencing (mNGS) of thoracic vertebral puncture tissue performed on December 3, 2025 detected *M. kansasii* (109 reads) and HBV (97,961 reads), further supporting disseminated infection with skeletal involvement. On December 10, 2025, he underwent anterolateral thoracic fusion with posterior pedicle-screw fixation, bone grafting, laminoplasty, and resection of the vertebral lesion. After surgery, the patient developed progressive liver dysfunction, characterized predominantly by cholestatic hyperbilirubinemia. On December 22, 2025, total bilirubin was 186.13 μmol/L, direct bilirubin 106.4 μmol/L, alanine aminotransferase 80 U/L, and aspartate aminotransferase 114 U/L; on December 25, 2025, total bilirubin increased to 231.45 μmol/L, direct bilirubin to 126.7 μmol/L, alanine aminotransferase to 91 U/L, and aspartate aminotransferase to 135 U/L. Serum HBV DNA was 6.23 × 10^6 IU/mL.

**Figure 3:**
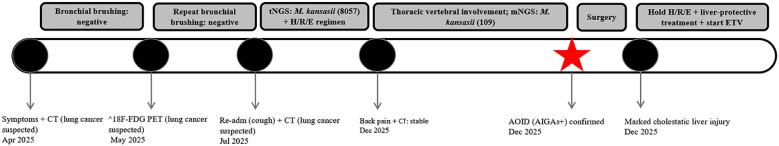
Timeline of the diagnostic and clinical course. BALF, bronchoalveolar lavage fluid; CT, computed tomography; ETV, entecavir; FDG, fluorodeoxyglucose; H/R/E, isoniazid/rifampin/ethambutol; LI, liver injury; mNGS, metagenomic next-generation sequencing; PET/CT, positron emission tomography/computed tomography; tNGS, targeted next-generation sequencing; Tx, treatment; AOID, adult-onset immunodeficiency; AIGAs, anti–interferon-γ autoantibodies.

Because disseminated *M. kansasii* infection is uncommon in apparently immunocompetent adults, additional immunologic testing using a serum anti–IFN-γ IgG enzyme-linked immunosorbent assay (ELISA) at Wuhan Kindstar Medical Laboratory revealed elevated anti–IFN-γ autoantibodies (IgG 106.19 RU/mL; reference range, 0–50 RU/mL). In the context of disseminated nontuberculous mycobacterial infection involving pulmonary and osseous sites, this finding supported a clinical diagnosis of anti–IFN-γ autoantibody-associated adult-onset immunodeficiency. Follow-up contrast-enhanced chest CT in January 2026 showed persistent soft-tissue density at the orifice of the left upper lobe bronchus, without significant regression compared with the July 2025 scan (**Figures 1H–L**). In view of the severe liver injury and bleeding risk, hepatoprotective and supportive treatment was administered, including ademetionine 1000 mg once daily, magnesium isoglycyrrhizinate 200 mg once daily, and acetylcysteine 8 g once daily. Entecavir was initiated for chronic HBV infection. Antimycobacterial therapy was temporarily withheld, and immunomodulatory treatment was deferred because of marked cholestatic liver dysfunction and high-level HBV viremia (**Figure 3**).

## Discussion

In this report, we describe a case of AIGA-associated disseminated *M. kansasii* infection involving both the lung and skeleton. A notable feature of this case was that the initial clinical and radiologic presentation closely mimicked primary lung cancer with multiple bone metastases, leading to a prolonged malignancy-directed diagnostic workup. The diagnosis was ultimately clarified only after repeated pathological examinations failed to confirm malignancy, and next-generation sequencing together with immunologic testing redirected the diagnostic pathway. This case highlights the importance of considering opportunistic infection and underlying immune dysregulation in adults with tumor-like multisystem lesions when conventional pathology remains inconclusive.

A major reason for the delayed diagnostic clarification in this case was the misleading radiologic presentation. The patient presented with a left hilar mass, segmental bronchial stenosis, and extensive FDG-avid skeletal lesions involving both the axial and appendicular skeleton. Taken together, these imaging findings initially directed the clinical workup toward a malignant process. In addition, the presenting symptoms of cough and chest pain further strengthened the clinical suspicion of lung cancer with bone metastases (7). However, increased ^18F-FDG uptake is not specific to malignant tissue. Activated inflammatory cells, including leukocytes, histiocytes, plasma cells, lymphocytes, and macrophages, may also show prominent tracer accumulation because of increased glucose uptake and accelerated glycolysis (8). Accordingly, a variety of infectious, inflammatory, and granulomatous conditions can yield positive PET/CT findings and radiologically mimic malignancy (9, 10). In this setting, FDG uptake should be interpreted together with lesion distribution, interval changes, pathological findings, and host immune status. In our patient, the lung mass and multifocal skeletal uptake initially favored metastatic lung cancer, but repeated negative malignancy-directed sampling and the relative radiologic stability of the pulmonary lesion were not fully consistent with this impression. The subsequent detection of *M. kansasii* in vertebral tissue further supported a diagnostic shift toward disseminated infection. When PET/CT findings strongly suggest metastatic malignancy but histopathologic evidence remains repeatedly negative, infectious etiologies should be reconsidered promptly.

As a clinically relevant infectious mimic in this setting, *M. kansasii* is the sixth most frequently isolated NTM species worldwide, accounting for approximately 9.4% of NTM isolates (11). The clinical presentation of *M. kansasii* infection often resembles that of *Mycobacterium tuberculosis*, making differentiation based on symptoms and imaging findings challenging. Common initial symptoms include cough, chest pain, minor hemoptysis, and dyspnea (12). Extrapulmonary involvement is relatively rare in the general population. A large study by Kaustová et al. found that only 0.6% of patients with *M. kansasii* infection developed extrapulmonary manifestations (13), whereas a survey from the UK reported extrapulmonary involvement in 8%–9% of cases (14). However, in the setting of impaired immune function, the risk of disseminated infection is significantly increased, and anti–IFN-γ autoantibody-associated immune abnormalities have been identified as an important susceptibility factor. Chi et al. reported that the prevalence of AIGA among patients with disseminated NTM infection reached 97.8% (15). Therefore, in HIV-negative patients with disseminated nontuberculous mycobacterial infection, anti–IFN-γ autoantibody testing should be considered to identify possible underlying AIGA.

We further reviewed the published cases of AIGA-associated *M. kansasii* infection with available clinical information and summarized them in **Table 1** (16–23). A total of eight previously reported cases were identified, published between 2013 and 2025. Clinical characteristics, including presenting symptoms, osseous involvement, tumor-like manifestations, treatment regimens, and outcomes, were extracted. As shown in **Table 1**, the clinical presentation was heterogeneous, including fever, cough, dyspnea, lymphadenopathy, skin or soft-tissue lesions, and back or bone pain. Osseous involvement was reported in different forms, including bone marrow involvement without focal bone lesions, multifocal skeletal lesions on bone scintigraphy or PET/CT, osteosclerotic or osteolytic changes, and destructive lesions involving the ribs, spine, pelvis, or other skeletal sites. Tumor-like or proliferative disease-like manifestations were also frequent. Several cases were initially suspected as multicentric Castleman disease, Rosai–Dorfman disease, POEMS/TAFRO syndrome, multiple myeloma, lung cancer with obstructive pneumonia, or metastatic disease. Most patients improved after antimycobacterial therapy, although treatment regimens and adjunctive therapies varied among reports. One fatal case with rapid clinical deterioration was also reported (16). Because of the small number of cases and the heterogeneity of infection sites, concomitant infections, and treatment strategies, outcomes could not be reliably compared between patients with and without osseous involvement. Nevertheless, these reports suggest that bone or bone marrow involvement is not rare in disseminated AIGA-associated *M. kansasii* infection and that tumor-like imaging or clinical presentations may delay the correct diagnosis.

**Table 1:** Summary of reported cases of AIGA-associated Mycobacterium kansasii infection.

Year and author	Main complaint or presentation	Osseous involvement	Tumor-like manifestation	Treatment	Outcome
Nei et al., 2013 (16)	Dyspnea, anemia, marked leukocytosis, and mediastinal lymphadenopathy; initially suspected as multicentric Castleman disease.	Bone marrow involvement; no focal bone lesion was reported.	Yes; suspected multicentric Castleman disease/lymphoproliferative disorder.	Tocilizumab and corticosteroids before diagnosis; isoniazid, rifampicin, and ethambutol after *M. kansasii* identification.	Clinical deterioration with DIC and multiple organ failure; died on hospital day 48.
King et al., 2017 (17)	Fever of unknown origin, painful cervical/supraclavicular masses with purulent discharge, and multiple tender erythematous cutaneous nodules; CT showed multifocal soft-tissue masses and lymphadenopathy.	Yes; multifocal bone lesions were shown on Tc-99m MDP bone scan and regressed after treatment.	Yes; mimicked Rosai–Dorfman disease with RDD-like histopathological features.	Clarithromycin, rifampicin, and ethambutol for 14 months.	Skin lesions almost completely resolved within one month; bone lesions regressed, with no recurrent skin lesions during follow-up.
Kashihara et al., 2019 (18)	Three-week history of cough and fever; right top-lobe consolidation with supraclavicular, mediastinal and hilar lymphadenopathy.	Yes; right costal skeletal lesion was suggested by Tc-99m skeletal scintigraphy.	No definite tumor-like manifestation reported; initially suspected as tuberculous lymphadenitis rather than malignancy.	Initially treated with isoniazid, rifampicin, ethambutol, and pyrazinamide; pyrazinamide was discontinued after *M. kansasii* identification, with short-course corticosteroid for suspected paradoxical reaction.	Symptoms improved over two months; patient remained well during outpatient follow-up.
Hidekawa et al., 2022 (19)	Polyarthralgia and multiple cellulitis, with POEMS/TAFRO-like systemic manifestations including anasarca, organomegaly, skin pigmentation, polyneuropathy, thrombocytopenia, serum M protein and reticulin fibrosis.	Yes; osteosclerotic and osteolytic bone changes were reported.	Yes; mimicked POEMS and TAFRO syndromes.	Anti-NTM therapy with rifampicin, ethambutol and clarithromycin, later changed to rifampicin, ethambutol and isoniazid; combined with glucocorticoid and IVIG.	Symptoms improved.
Pan et al., 2023 (20)	Tender erythematous subcutaneous nodules on the chin and neck, with recurrent fever, lymphadenopathy and pulmonary lesions.	Yes; PET-CT showed FDG-avid multiple skeletal lesions.	No explicit malignancy-mimicking presentation was reported; PET-CT showed FDG-avid lymphadenopathy, pulmonary lesions and skeletal lesions.	Rifampicin, clarithromycin and moxifloxacin.	Fever resolved, skin ulcers healed and lymph nodes narrowed; no recurrence at 6-month follow-up.
Wang et al., 2023 (21)	Recurrent pneumonia and pulmonary lesions, followed by fever, headache, lower back pain and progressive bone destruction.	Yes; multifocal bone destruction involving the ribs, spine and pelvis, with *M. kansasii* detected from bone marrow puncture fluid.	Yes; bone lesions were suspected as multiple myeloma, with PET-CT showing hypermetabolic lymph nodes and multifocal skeletal lesions.	Antifungal therapy for *T. marneffei*, antibacterial therapy for *L. monocytogenes*, and anti-NTM therapy with clarithromycin, rifampicin, ethambutol and linezolid.	Symptoms, pulmonary lesions and bone destruction improved; no discomfort and normal inflammatory markers at follow-up.
Chou et al., 2023 (22)	Fever, productive cough, weight loss and severe dyspnea; initially suspected as lung cancer with obstructive pneumonia.	Bone marrow involvement; no focal osseous lesion reported.	Yes; initially suspected as lung cancer with obstructive pneumonia.	Azithromycin, moxifloxacin and rifampicin for 12 months; no immune-modulating treatment.	Symptoms improved, imaging abnormalities resolved, and no recurrent NTM infection was noted after treatment cessation.
Wei et al., 2025 (23)	Persistent cough, back pain, fever, generalized pustular rash and progressive lymphadenopathy.	Yes; Tc-99m skeletal scintigraphy showed multifocal osteoblastic activity.	Yes; right hilar mass and skeletal lesions raised suspicion of metastatic disease, but biopsy showed no malignancy.	Isoniazid, clarithromycin, moxifloxacin and linezolid for NTM; amphotericin B followed by itraconazole for *T. marneffei*.	Favorable clinical outcome with improvement of pulmonary lesions, hilar mass and lymphadenopathy.

AIGA, anti-interferon-gamma autoantibody; CRP, C-reactive protein; CT, computed tomography; DIC, disseminated intravascular coagulation; FDG, fluorodeoxyglucose; IVIG, intravenous immunoglobulin; MDP, methyl diphosphonate; NTM, nontuberculous mycobacteria; PET-CT, positron emission tomography-computed tomography; POEMS, polyneuropathy, organomegaly, endocrinopathy, monoclonal plasma cell disorder and skin changes; RDD, Rosai–Dorfman disease; TAFRO, thrombocytopenia, anasarca, fever, reticulin fibrosis/renal dysfunction and organomegaly; Tc-99m, technetium-99m; *M. kansasii*, *Mycobacterium kansasii*; M. smegmatis, *Mycobacterium smegmatis*; T. marneffe*i*, *Talaromyces marneffei*; *L. monocytogenes*, *Listeria monocytogenes*.

From a therapeutic perspective, management of *M. kansasii* infection in the setting of AIGA generally involves two complementary components: antimycobacterial therapy and immune-directed treatment. According to the ATS/ERS/ESCMID/IDSA guideline, rifampin-susceptible *M. kansasii* should be treated with a rifampin-based multidrug regimen, usually in combination with ethambutol and isoniazid, for at least 12 months (24). However, in patients with AIGA, treatment is often more challenging than in immunocompetent hosts because persistent immune dysfunction predisposes to disseminated disease, recurrent infection, and infection with newly emerging or drug-resistant pathogens. In clinical practice, these patients frequently require a longer treatment course, often extending to 18–24 months (5, 25). According to the available literature, the reported relapse and mortality rates are 21.62% and 11.68%, respectively (5). In addition to antimicrobial therapy, immunomodulatory agents such as rituximab and cyclophosphamide have been used to reduce autoantibody-mediated immune dysfunction. Nevertheless, the optimal antimicrobial regimen, treatment duration, and criteria for treatment discontinuation in AIGA-associated *M. kansasii* infection remain to be established. In our patient, further treatment escalation was constrained by postoperative cholestatic jaundice and chronic HBV infection with high-level viremia. Under these circumstances, immediate initiation of rituximab-based immunotherapy was considered high risk, given the well-recognized association between rituximab and HBV reactivation (26). Accordingly, antiviral therapy and hepatic stabilization were prioritized before reconsideration of immune-directed treatment. Likewise, because jaundice and clinically significant liver injury generally warrant interruption of potentially hepatotoxic antimycobacterial agents, the rifampin- and isoniazid-containing regimen was temporarily withheld, and subsequent treatment adjustment was planned according to liver function recovery and drug tolerance.

Limited short-term radiologic regression despite antimycobacterial therapy was observed in this case. A previous case report described a patient with *M. kansasii* pulmonary infection presenting with a left lung mass, in whom the lesion showed interval regression after 6 months of rifampin-based antimycobacterial therapy, without evidence of underlying AIGA (27, 28). By contrast, the absence of obvious shrinkage in our patient may have been related not only to the short treatment period but also to persistent underlying immune dysregulation. In this context, the lack of obvious early shrinkage was clinically understandable.

The additional BALF tNGS findings were interpreted in the overall clinical context. Besides *M. kansasii*, BALF tNGS also detected *Pneumocystis jirovecii*, Epstein–Barr virus, *Streptococcus pneumoniae*, and *Burkholderia neonosa*. Among these organisms, only *M. kansasii* was also identified in vertebral tissue, making it more consistent with the pulmonary lesion and multifocal osseous involvement. The patient did not show typical clinical or imaging features of Pneumocystis pneumonia, and there was no clear clinicoradiologic evidence supporting *Burkholderia* as the main cause of the disseminated lesions. Therefore, the additional BALF findings were considered more likely to represent airway colonization, concomitant respiratory organisms, or viral reactivation rather than the main driver of the disease. EBV-associated lymphoproliferative disease, including lymphomatoid granulomatosis, was also considered, but repeated cytologic and pathologic examinations did not support malignant or atypical lymphoid proliferation.

This report has several limitations. As a single case, it cannot define the full clinical spectrum of AIGA-associated disseminated *Mycobacterium kansasii* infection. Conventional mycobacterial culture and drug susceptibility testing were not performed, which limited culture-based confirmation and susceptibility-guided treatment adjustment. In addition, more comprehensive immunological follow-up, including serial anti–IFN-γ autoantibody measurements, HLA typing, and functional neutralization assays, was not available, which limited further characterization of the underlying immune dysfunction. Long-term treatment response could not be fully assessed because antimycobacterial therapy was interrupted and immune-directed therapy was deferred.

## Conclusion

In adults with tumor-like pulmonary lesions and multifocal skeletal abnormalities, repeatedly negative pathology should prompt reconsideration of infectious mimics, particularly when lesions involve multiple organs. For suspected disseminated NTM infection, pathogen detection should be pursued from clinically involved sites, and common immunocompromising conditions should be assessed. When disseminated NTM infection occurs in an HIV-negative adult without an obvious immunosuppressive condition, anti–IFN-γ autoantibody testing should be incorporated early into the diagnostic workup. This approach may help distinguish AIGA-associated opportunistic infection from metastatic malignancy and reduce diagnostic delay.

## Data Availability

The original contributions presented in the study are included in the article/supplementary material. Further inquiries can be directed to the corresponding author.
